# Cognitive cost as dynamic allocation of energetic resources

**DOI:** 10.3389/fnins.2015.00289

**Published:** 2015-08-24

**Authors:** S. Thomas Christie, Paul Schrater

**Affiliations:** ^1^Cognitive Science, University of MinnesotaMinneapolis, MN, USA; ^2^Departments of Psychology and Computer Science, University of MinnesotaMinneapolis, MN, USA

**Keywords:** decision making, optimal control, glycogen, energy, cognitive control, mental effort

## Abstract

While it is widely recognized that thinking is somehow costly, involving cognitive effort and producing mental fatigue, these costs have alternatively been assumed to exist, treated as the brain's assessment of lost opportunities, or suggested to be metabolic but with implausible biological bases. We present a model of cognitive cost based on the novel idea that the brain senses and plans for longer-term allocation of metabolic resources by purposively conserving brain activity. We identify several distinct ways the brain might control its metabolic output, and show how a control-theoretic model that models decision-making with an energy budget can explain cognitive effort avoidance in terms of an optimal allocation of limited energetic resources. The model accounts for both subject responsiveness to reward and the detrimental effects of hypoglycemia on cognitive function. A critical component of the model is using astrocytic glycogen as a plausible basis for limited energetic reserves. Glycogen acts as an energy buffer that can temporarily support high neural activity beyond the rate supported by blood glucose supply. The published dynamics of glycogen depletion and repletion are consonant with a broad array of phenomena associated with cognitive cost. Our model thus subsumes both the “cost/benefit” and “limited resource” models of cognitive cost while retaining valuable contributions of each. We discuss how the rational control of metabolic resources could underpin the control of attention, working memory, cognitive look ahead, and model-free vs. model-based policy learning.

## 1. Introduction

Cognitive processes that require vigilance, model-based lookahead, or extensive utilization of attention or working memory are said to incur a cost (Kool et al., 2010), described as aversive (McGuire and Botvinick, 2010), or characterized as computationally expensive (Redish, 2013). General avoidance of such processes makes us “lazy organisms” (McGuire, 1969) and “cognitive misers” (Taylor, 1981), who in many circumstances would rather use “fast and frugal” heuristics (Gigerenzer and Goldstein, 1996) or habits (Redish, 2013) than “intrinsically costly” deliberative thought (Kool and Botvinick, 2014). Echoing Solomon's “law of least effort” (Solomon, 1948), Balle suggested that humans follow a “law of least mental effort” (Balle, 2002), always seeking the least cognitively expensive way of achieving a goal.

Such claims raise the question of *why* certain types of cognition are costly. What, exactly, is being spent? Botvinick and colleagues have suggested that cognitive leisure has inherent value, and that we forego this value by engaging in cognitive laborious processes (Kool and Botvinick, 2014). A recent paper by Kurzban et al. (2013) proposes that focusing limited cognitive resources on a single task to the exclusion of other possible tasks carries an opportunity cost. In this model, a subjective feeling of fatigue is a signal to switch to more worthwhile tasks. Kool et al. (2010) propose that the avoidance of cognitive demand is a fundamental principle of cognition, though the authors fail to specify why cognition should be demanding. These approaches are sometimes called economic or “cost/benefit” models of cognitive cost, as they model subject behavior as attempting to optimally trade costs (leisure, opportunity cost, cognitive demand) for benefits (reward, leisure).

“Cost/benefit” models provide an explanation for the finding that increasing reward in specific types of tasks can induce subjects to exert more cognitive effort (Camerer and Hogarth, 1999; Jimura et al., 2010) and improve executive function (Krebs et al., 2010). An pernicious drawback of “cost/benefit” models is that they decouple the act of cognition from its fundamentally limited biological substrate, the brain. The models mentioned here treat cognition as bounded or restricted, which is equivalent to assuming that cognition has a limited bandwidth but unlimited resources. As such, they are unable to convincingly account for the *dynamics* of cognitive exertion and mental fatigue. “Cost/benefit” models are also fundamentally divorced from the well-established connection between blood sugar levels and cognitive function, a relationship familiar to every doctor and diabetic. As the blood sugar level of an individual falls, precisely those processes that are considered costly are first affected (see Feldman and Barshi, 2007).

Another influential approach links cognitive costs to the utilization of a limited resource, purportedly blood glucose (Gailliot et al., 2007). In this model, use of costly processes diminishes available glucose, leaving less fuel for future processes. Proponents of this assumption typically focus on self-control, one instantiation of top-down executive control over behavior. In the frequently used “dual-task” paradigm, subjects are asked to perform an initial “depleting” task involving extensive use of self-control. They are then asked to perform a second demanding task. A commonly reported finding is that subjects perform worse on the second task *only if* the first task was sufficiently demanding, and the performance decrement can be eliminated by offering subjects a glucose drink (see e.g., Gailliot et al., 2007). Unlike “cost/benefit” models, “limited resource” models offer no mechanism by which motivational factors can influence subject behavior. Additionally, reported blood sugar decreases arising from cognitively demanding tasks (as reported by Fairclough and Houston, 2004; Gailliot et al., 2007) are much smaller than the changes required to effect task performance (see below). Finally, the strength of “limited resource” findings are coming under increasing scrutiny from *post-hoc* analyses (Hagger et al., 2010; Carter and McCullough, 2014).

In the present article we introduce a new model that treats cognitive resources as depending squarely on a metabolic substrate with explicitly specified dynamics, while still allowing for the possibility of motivational factors to alter agent performance. We suggest that an individual's decision of whether or not to incur cognitive costs in a given situation can be fruitfully understood as one of decision making *strategy*: an agent will only commit limited resources in cases where the payoff is worth it. Unlike “cost/benefit” models, however, we treat resources as dynamically utilized and replenished. Much like a marathon runner, an agent attempting to optimize long-term performance may choose to purposefully limit exertion in order to maintain resource reserves for future use. What may *appear* to be aversion to cognitive effort may in fact be strategic resource allocation.

A view of the brain as engaging in cognitive strategy selection that is dynamic, constrained, and maximizing an objective function is naturally modeled using optimal control theory. Using an optimal control theory framework allows and requires the modeler to be explicit about the dynamics of the system being modeled, the objective function to be optimized, and the planning horizon of the agent. From this perspective, assumptions of limited resources and cost/benefit tradeoffs are not diametrically opposed, as some have suggested (Kurzban et al., 2013), but are different components in a more general framework. So-called “opportunity costs” are simply a special case in which an agent has more than one task available to choose from. The “limited resource” assumption is a special case in which system dynamics are specified but in which no explicit claim is made about what is being optimized.

In what follows, we provide an optimal control model of energy use in the brain. The model provides a novel explanation of cognitive costs as arising from intelligent resource allocation over time. We briefly review evidence supporting our specification of system dynamics, objective function, and controls. We then discuss results from a computational implementation of our model, and compare the effects of various modeling assumptions. Finally, we discuss the implications of our model and suggest directions for future work.

## 2. Dynamic resource control

### 2.1. Overview

Optimal control theory is a mathematical approach to optimizing dynamic action selection. Given a system with intrinsic dynamics, a controller repeatedly receives signals from the system, estimates its state, and executes actions in order to optimize an objective function over time. We develop the hypothesis that the brain has an intelligent control system for managing its use of metabolic resources by trading off performance for reductions in neural activity. We first review the evidence for a control system view of energy management, and then development a mathematical model of the same.

#### 2.1.1. Cognitive fatigue and cost as energy depletion

Human cognition is a biological process operating within biological constraints. As such, it is not surprising that hypoglycemia is known to cause performance decrements in cognitive tasks. Moderate hypoglycemia, such as that arising from fasting, can impair cognitive performance short-term verbal (Martin and Benton, 1999) and spatial (Benton and Parker, 1998) memory, and the speed of mental computation (Benton and Sargent, 1992; Donohoe and Benton, 1999; Kennedy and Scholey, 2000). More severe hypoglycemia has been shown to negatively affect performance in the Stroop task (Evans et al., 2000), multi-choice reaction time tasks (Gold et al., 1995; Evans et al., 2000; Schächinger et al., 2003), the PASAT mental arithmetic task (Cox et al., 1993; Gold et al., 1995), digit span tasks (Holmes et al., 1983; Pramming et al., 1986), the Tail Making B task (Hoffman et al., 1989; Gold et al., 1995; McCrimmon et al., 1997), tracking performance (Hoffman et al., 1989; Schächinger et al., 2003), attentional tasks (Lobmann et al., 2000; McAulay et al., 2001), driving (Cox et al., 2000), auditory processing (McCrimmon et al., 1997), though some studies report contradictory findings (Holmes et al., 1986; Manning et al., 1990; Benton and Owens, 1993; McAulay et al., 2001) and high individual variation in effects (Hoffman et al., 1989; Evans et al., 2000). In general, performance in tests requiring only simple motor functions (like the Finger Oscillation Test and Finger Tapping Task) are not affected (Manning et al., 1990; Cox et al., 1993). Providing subjects a glucose drink after fasting can improve performance (Manning et al., 1990; Benton and Owens, 1993; Craft et al., 1994; Benton and Parker, 1998; Martin and Benton, 1999). Similar improvement was often not found when subjects were given a sweetened placebo in place of a glucose drink to raise blood sugar levels (Craft et al., 1994; Donohoe and Benton, 1999; Scholey et al., 2001). Some studies report decreased reaction time while others reported decreased accuracy. We suspect that these differences can be explained by specifics of task design and reward structure, though there is some evidence for an effect of individual differences during mental fatigue (Hoffman et al., 1989; Evans et al., 2000). See (Feldman and Barshi, 2007) for a thorough review on the effects of glucose levels on cognitive function.

Subjective mental fatigue and time-on-task can produce similar performance decrements to hypoglycemia (Lorist et al., 2000; Healy et al., 2004). As mentioned above, proponents of the “limited resource” account of cognitive costs observe that the dynamics of cognitive fatigue and performance degradation is consistent with the depletion of a limited resource. That resource is largely assumed to be blood glucose. However, direct measurement of blood sugar levels (Fairclough and Houston, 2004; Gailliot et al., 2007) and metabolic rate (Huang et al., 2012) during cognitively demanding tasks show effects that are too small to account for the detrimental effects on performance that occur during a hypoglycemic state. We suggest that this apparent contradiction can be explained by accounting for the energy storage and buffer mechanism provided by astrocytic glycogen (c.f. Gailliot, 2008), in which astrocytic glycogen is the limited, depletable resource. If this is the case, glycogen-dependent neural activity should suffer when glycogen is depleted, while extended cognitive load would have little or no effect on overall blood glucose levels.

#### 2.1.2. Brain glycogen as depletable energy resource

Glycogen is a storage form of glucose. In the human body glycogen is primarily found in liver and muscle cells, but a small amount also exists in astrocytes. It has been estimated that glycogen metabolism in astrocytes accounts for only 1–6% of energy use in the brain under normal conditions (Benington, 1995). Despite the small amount of glycogen utilization, the amount of glucose stored in brain glycogen is thought to be greater than the amount of non-glycogenic glucose in the brain (Gruetter, 2002, cited in Gailliot, 2008). Moreover, studies of both *in vivo* and *in vitro* glycogen metabolism show that its usage is critically linked to periods of neural stimulation.

**Figure 1:**
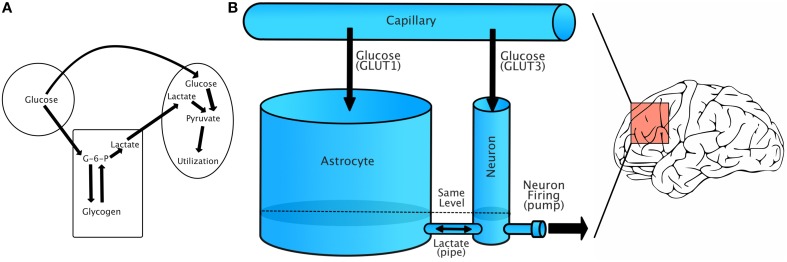
**Energy flow during increased demand. (A)** Simplified account of capillary/glycogen/astrocyte relationship, modified from Brown (2004). **(B)** A schematic of our model, in which energy is treated as a single fluid flowing between compartments. Lactate/GLUT labels are only for reference, as our model treats energy flow as the flow of a single fluid. The model should be understood to represent dynamics in a small region of the brain that has relatively uniform energy dynamics.

Figure 1A shows a simplified schematic of the relationship between capillaries, astrocytes, and neurons. Glucose from capillaries is transported to astrocytes and neurons via facilitative glucose transporter proteins (GLUT1 for astrocytes and GLUT3 for neurons). Glucose is phosphorylated to glucose-6-phosphate upon entering an astrocyte. From there, it can be either stored as glycogen or converted to lactate. Lactate is transported from astrocytes to neurons using monocarboxylate transporters (Simpson et al., 2007).

In a series of *in vitro* experiments, Brown and colleagues investigated the dynamic relationship between astrocytic glycogen and action potentials in the mouse optic nerve in the presence and absence of glucose (Brown et al., 2003). See **Figures 4A,C** for plots of the relevant dynamics. Axonal response to stimulation was measured using compound action potential area, or CAP area. When glucose was removed from the environment and neural tissue was stimulated, CAP area remained robust for up to 20 min, during which time glycogen stores in astrocytes decreased in a remarkably linear fashion. Glycogen content of astrocytes was strongly predictive of the duration of sustained activity following aglycemia (see **Figure 4C**). In addition, inhibiting monocarboxylate/lactate transfer caused CAP area to decrease sharply rather than remain stable, while introducing lactate sustained CAP area when glycogen was absent. These observations strongly indicate that astrocytic glycogen acts as a supplementary fuel reservoir for neuronal activity. Moreover, in the presence of normal glucose levels, axonal stimulation still led to a (less rapid) drop in astrocytic glycogen levels, indicating that glycogen is utilized even in the presence of a normal concentration of glucose. Finally, CAP area was maintained when lactate transport was blocked and a high concentration of glucose was present, but not when the concentration of glucose was low, indicating that neurons also use glucose directly (see Brown et al., 2003 Figure 3C, also Simpson et al., 2007).

The link between neuronal stimulation and glycogen utilization is supported at a larger scale by in research involving animal models. Choi et al. (2003) subjected rats to insulin-induced hypoglycemia. As brain glucose approached zero, brain glycogen content (as measured using 13-C NMR) declined gradually and sustained brain activity for 2 h. Glycogen utilization has also been shown to increase by tactile stimulation of rats (Swanson, 1992; Swanson et al., 1992; Dienel and Cruz, 2006).

Glycogen stores have been shown to increase during sleep (Swanson, 1992), anesthesia and sustained levels of high blood sugar (Nelson et al., 1968), and to decrease during sleep depravation (Karadzic and Mrsulja, 1969) and one-trial learning (Hertz et al., 1996). Glycogen accumulates faster in regions of the brain that have highest synaptic density (Phelps, 1972) and has a high concentration in the cerebellum, hippocampus, thalamus, and striatum (Sagar et al., 1987). Finally, glycogen does not appear to be a passive reservoir, utilized only when energy need exceeds resources. Glycogen can be synthesized and degraded simultaneously (Brown and Ransom, 2007), and glycogen turnover rate increases in the presence of nearby neural activity (Pentreath and Kai-Kai, 1982; Swanson et al., 1992).

Taken together, current evidence strongly suggests that astrocytic glycogen acts as a energy shunt or reservoir that is depleted during periods of high neural activity and repleted during rest, a shunt into which energy is consistently being deposited and released. This stored energy can support neural activity that exceeds the instantaneous resources of available glucose, and is replenished during rest.

### 2.2. Optimal control

An optimal control problem is fundamentally defined by system dynamics, controller sensors and an objective function. In the model we present, energy resources in the brain have intrinsic dynamics and represent the system to be controlled. The system state consists of the resources available in different components of the brain, namely the energy residing in astrocytes and neurons. The system dynamics describe energy flow between capillaries, astrocytes, and neurons within an area. Control of the system takes the form of increasing and decreasing energy usage rate. In neural terms, we interpret this control as the change in concentration of some excitatory neuromodulator. An increase in neuromodulator concentration increases neural excitability and the recruitment of more active neurons, with which task-related energy utilization increases linearly. Actual neural recruitment and associated energy utilization depends on available resources, so the energy flow out of neurons is a function of both the excitatory concentration and available energy. The population of recruited neurons maps to performance using a task-specific performance curve (see **Figure 3**).

In an optimal control problem, a controller attempts to optimize an objective function (also called an objective function) over time. We assume that the objective function includes rewards given for task performance. Whether the objective function should include cognitive costs (indeed, the very phrase “cognitive costs” belies the implication) is an open question. The received wisdom is that cognitive costs are something to be avoided for their own sake—that is, they belong in the objective function. We suggest the possibility that apparently “costly” cognitive processes are instead avoided because they strongly affect energy resource availability, and hence performance at longer time scales. If an agent has the capacity to plan, they may judiciously use resources in order to maximize reward over time. Indeed, the model we propose herein suggests a method for discerning whether cognitive costs can be said to exist *per se* (see Section 3.5).

Our proposed mapping between the optimal control framework and brain energy dynamics is given in Table 1. The goal of the controller is to execute a sequence of control actions that minimizes the objective function over a given time horizon. The objective function *J*, shown in Equation (1) for a time horizon *n* and initial state *x*_0_, is *additive* in that it is the sum of costs at each time step *k*.

(1) $$\begin{array}{l} J\left(x_{0}\right)=E\left\{\overset{n}{\underset{k=0}{\sum }}g\left(x_{k},u_{k}\right)\right\} \end{array}$$

**Table 1:** **Proposed relationship between a control theoretic framework and the optimization of energy use in the brain over time**.

**Control theory**	**Variable**	**Interpretation**
Time index	*k*	Index of time in a scale in which distinct control actions are possible
State	*x*_*k*_ = [*H*_*N*_, *H*_*A*_]	Quantity of energy available to neurons and astrocytes
Control	*u*	Neuromodulator concentration driving neural recruitment in an area
Dynamics	*x*_*k* + 1_ = *f*(*x*_*k*_, *u*_*k*_)	Energy dynamics in the brain
Objective function	*g*(*x*_*k*_, *u*_*k*_)	Combination of cognitive costs (if any) and rewards for a single time step
Objective function	*J*	Overall objective function to be optimized over a given time horizon

Energy dynamics operate at varying temporal and spatial scales. The model we introduce in the present work is intended to represent neural dynamics on the time scale of roughly 20 min to several hours, as that is the time scale of glycogen depletion and subjective mental fatigue. The spatial scale of our model is a local brain region with homogenous energy resources, subserving some particular cognitive function. Elsewhere in this paper, we consider cognitive functions like working memory, executive control, and attention to be candidate functions as they are sensitive to both energetic resources and reward, but in the present model we treat only a simplified cognitive function whose utilization maps to performance in a one-dimensional manner. This function is intended to be the simplest case of a energy- and reward-sensitive cognitive function, and more investigation is needed to understand how specific functions like attention or working memory should be modeled. We envision energy dynamics being regulated across several regions (and functions) at longer time scales, however, here our goal is to develop an area specific control model whose extension to the full brain via hierarchy and composition is the subject of future work.

We model the task-related dynamics of energy use using a compartmental fluid flow model and show that our model is consistent with available data. We then show how neural activity can be modulated to control metabolic usage and maximize long-term task performance.

#### 2.2.1. Dynamics model

We use glycogen-supported energy transfer as a dynamics model in our optimal control approach to modeling cognitive costs. As we are at present introducing a model that we hope is *qualitatively* useful, we will use a greatly simplified model of energy flow from capillaries to astrocytes to neurons. Astrocytic glycogen and glucose is treated as one energy pool, and neuronal lactate and glucose is treated as another.

The model we propose is illustrated in Figure 1B. In this model, energy (in the form of glucose) flows from capillaries to both astrocytes and neurons. We model energy flow as a fluid flowing from capillaries into astrocyte and neuron “buckets” connected by a pipe. Though molecule transfer occurs via transporter-facilitated diffusion, we make a simplifying assumption and model the transfer of energy as the flow of a liquid though containers, constrained only by the “radius” of connecting pipe. Liquid flow between the two buckets is governed by the Torricelli Model, in which the change in height of the fluid in a bucket is proportional to the square root of the fluid height. Each bucket is treated as “leaky,” leaking into the other bucket.

The fluid flows from a capillary reservoir to both neurons and astrocytes at a certain rate. Astrocytes are modeled as having a greater energy/fluid capacity than neurons. Energy can flow freely from astrocytes to neurons and back via a pipe at the base of both containers (this models lactate transfer between cells). Fluid levels naturally move toward equilibrium in connected vessels. Energy/fluid can flow out of neurons via a variable rate active pump. The requested rate of pumping, or energy utilization, is the single control variable of the system.

**Figure 2:**
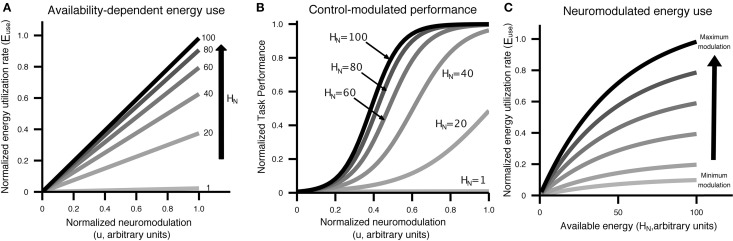
**Visual display of the relationship between control/neuromodulation (*u*), energy availability (*H*_*N*_), energy use (*E*_*use*_), neural recruitment [*NeuralRecruit*(*x, u*)], and performance [*Perf*(*x, u*)]**. Curves were generated using the equations from Equation (5) and parameters from Table 2. **(A)** The relationship between control/neuromodulation and energy utilization is modeled as linear, with the slope controlled by the amount of energy available to neurons. **(B)** Performance as a function of control/neuromodulation on a specific task at different levels of energy availability. This figure illustrates that the same performance can be achieved with different levels of available energy through the application of varying levels of control/neuromodulation. **(C)** Energy utilization has an upper bound and is affected by both control/neuromodulation and energy availability.

The modeled relationship between requested energy utilization and actual utilization is not linear. If the level of energy in a neuron is zero, clearly no energy is present to be utilized. Inspired by dynamics illustrated in Brown et al. (2003), we model the relationship between requested energy utilization and actual energy flow as a bounded exponential. The actual energy utilization rate depends on both the energy level in the neuron and the requested rate. This relationship is illustrated in Figure 2. The complete state dynamics are given by Equation (2):

(2) $$\begin{array}{l} \begin{array}{l} x_{k+1}=f\left(x_{k},u_{k}\right)\\ x_{k+1}\approx x_{k}+\frac{dx}{dt}\left(x_{k},u_{k}\right)\Delta t \end{array} \end{array}$$

where *dx*/*dt* is the differential change in the state given by Equation (3):

(3) $$\begin{array}{r} \frac{dx}{dt}=\left[\begin{array}{c} dH_{A}/dt\\ dH_{N}/dt \end{array}\right]=\left[\begin{array}{c} \text{Between-compartment flow}+\text{Inflow from capillaries}\\ \text{Between-compartment flow}+\text{Inflow from capillaries}-\text{Outflow} \end{array}\right]\\ =\left[\begin{array}{c} \frac{a_{connector}}{a_{A}}\sqrt{\left|H_{N}-H_{A}\right|}\cdot \mathrm{sgn}\left(H_{N}-H_{A}\right)+\alpha E_{B}\\ \frac{a_{connector}}{a_{N}}\sqrt{\left|H_{A}-H_{N}\right|}\cdot \mathrm{sgn}\left(H_{A}-H_{N}\right)+\beta E_{B}-E_{use}\left(u,H_{N}\right) \end{array}\right] \end{array}$$

The energy outflow *E*_*use*_ is a function of energy demands due to the neural excitability control level *u* and the current neural energy availability *H*_*N*_.

(4) $$\begin{array}{r} E_{use}\left(u,H_{N}\right)=\gamma u\left(1-e^{-\delta H_{N}}\right) \end{array}$$

Each of the model parameters has a simple interpretation:

- *H*_*A*_, *H*_*N*_: Amount of energy in astrocyte and neuronal compartments, respectively modeled as fluid heights.
- *u*: energy outflow “demand” due to neural activity. This is akin to neuromodulator-induced neural recruitment in the brain, modulating energy use with task demands.
- *E*_*use*_(*u, H*_*N*_): energy outflow rate as a function of demand and availability. This relationship is modeled as a bounded exponential to account for both a zero rate of flow for an empty compartment and an upper bounded of energy utilization.
- *a*_*A*_, *a*_*N*_: energy capacity of astrocytes and neurons, respectively.
- *a*_*connector*_: parameter controlling the energy flow rate between the astrocyte and neuron compartments.
- α, β: parameters controlling the energy flow rate from the capillary to the astrocyte and neuronal compartments, respectively.
- γ: scaling parameter controlling the utilization of energy as a function of neuron energy level.
- δ: parameter controlling the sensitivity of energy use to low energy availability.
- *E*_*B*_: energy content of the blood. Used to modulate the overall flow of energy into the neuron and astrocyte buckets and simulate hyper- and hypo-glycemia.

#### 2.2.2. Cost model

The goal of the controller is to optimize the objective function over a given time horizon. Each time step *k* is an instance at which controls can be executed. The time scale is intrinsically set by the system dynamics. In our case, it is the time scale at which an agent can change its level of cognitive effort. This is not necessarily the same as the time scale of a given trial-based task; *k* does not correspond to the trial number. The time scale at which individuals can ramp up or down cognitive effort is a factor that should be measured empirically, and should not be assumed to be dictated by a given task.

**Figure 3:**
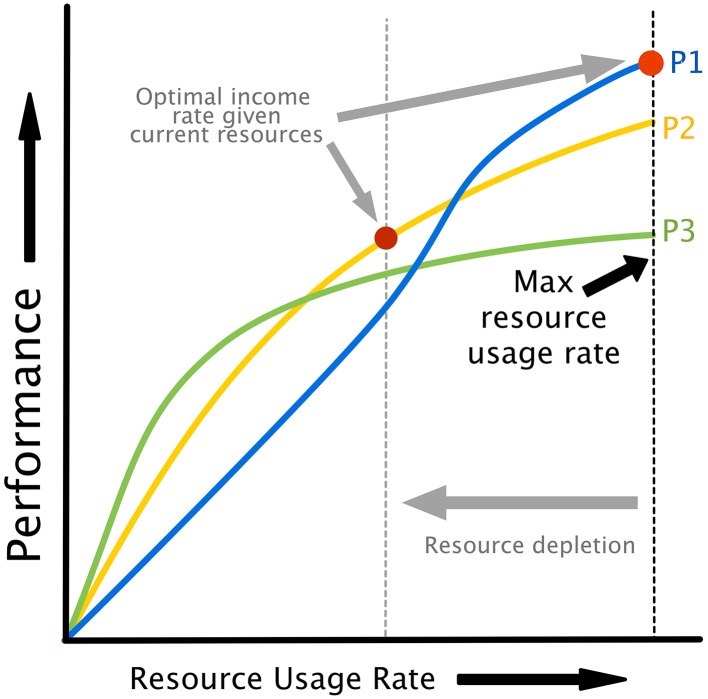
**Performance curves illustrating the relationship between resource utilization and performance**. Different curves can represent either different policies/strategies for the same task or different tasks. Note that as resources are depleted, the maximum possible energy utilization rate drops (see Figure 2C), creating the possibility of a different strategies or task being optimal based on energy dynamics alone. This provides a natural explanation for task or strategy switching as a result of fatigue.

The relationship between attempted cognitive control *u*, resource availability [*H*_*N*_, *H*_*A*_] and performance is illustrated in Figure 2B. Cognitive control is mapped to task performance in a two step process. First, requested control is converted into actual energy utilization in a fashion that depends on the amount of energy available to neurons. Energy utilization is assumed to reflect neural recruitment for a particular task, which could affect working memory load, attention, vigilance, or any other cognitive resources that are variable and contribute to task performance. Then, energy utilization is mapped to performance through a monotonically increasing, task-specific performance curve. Example performance curves are shown in Figure 3. Each colored line represents a possible relationship between resource utilization and performance for a given task, or different strategies on a single task.

Our model assumes that resources can be utilized at a rate dependent on the available energy level. If different tasks or strategies are available as in Figure 3, an agent might change tasks or strategies (or “policies,” in the terminology of optimal decision making) in order to maintain optimal performance. The availability of a set of performance curves dependent on resource levels, whether they represent tasks or different strategies for the same task, provides a natural explanation for task and strategy switching that does not rely on theoretical constructions like “opportunity costs.”

Equations (5) represent the control-to-performance relationship at time step *k*. The cost *g*(*x, u*) for a state-control pair is calculated to be a linear combination of the control *u* and task performance (which is assumed to translate directly to reward).

(5) $$\begin{array}{l} \begin{array}{ll} g\left(x,u\right) & =\lambda u-Perf\left(x,u\right)\\ Perf\left(x,u\right) & =\frac{1}{1+e^{-NeuralRecruit\left(x,u\right)}}\\ NeuralRecruit\left(x,u\right) & =\gamma_{e}E_{use}\left(x,u\right)+\gamma_{f}\\ E_{use}\left(x,u\right) & =\delta u\left(1-e^{-\gamma H_{N}}\right) \end{array} \end{array}$$

where *Perf* is the performance curve relating neural recruitment and energy consumption to task performance, *NeuralRecruit* represents the population size recruited given the energy use. Parameter values in γ_*e*_ and γ_*f*_ were chosen to make the variable part of *Perf* have the same scale as the glucose usage *E*_*use*_. The control component of this sum is represented by the λ*u* term.

When λ > 0, the control variable *u* influences the objective function, in which case cognitive control can be said to be intrinsically costly. While we expressed costs in terms of control (which is traditional for optimal control theory), we could have equivalently used the linear relationship between *u* and energy use, to express the costs in terms of energy use, or nonlinearly in terms of neural recruitment. Thus, λ represents both a gain on control cost and a unit conversion factor. We explore the implications of λ-values in Section 3 below.

### 2.3. Model discussion

Energy dynamics in the brain can be understood to operate at several temporal and physical scales. At the smallest scale, glutamate must be rapidly (≈ 10 ms, see Bergles and Jahr, 1997) sequestered following firing to prevent glutamate toxicity. This operation is performed much more quickly than increased regional cerebral blood flow and is thought to be powered by the rapid utilization of astrocytic glycogen (see Shulman et al., 2001). At the scale of minutes and hours, high levels of neural activity deplete glycogen stores (see above). This is the scale at which our model is focused. Blood glucose concentration is affected by exercise and food consumption. In our model, the flow of energy from capillaries to astrocytes directly affects the replenishment rate of glycogen (modeled as energy level in the astrocyte bucket). Flow from capillaries to astrocytes and neurons limits the maximum neuronal firing rate following glycogen depletion, in which case energy is utilized as soon as it enters the neuron bucket from the astrocyte bucket or capillary.

As mentioned above, the proposed model allows for a simple explanation of activity-induced cognitive deficits reported in the ego depletion literature. Baumeister and colleagues propose a resource-depletion framework in which self-control (and possibly other cognitive functions) deplete a limited resource (Gailliot et al., 2007). They suggest that this resource may be blood glucose (Gailliot et al., 2007), citing the common finding that a meal or glucose drink eliminates cognitive or self-control deficits that follow a demanding task. Instead, we propose that it is *glycogen* stores that are depleted in the presence of sustained cognitive effort. In the absence of increased blood sugar levels, performance should suffer following glycogen depletion. On the other hand, increasing blood sugar levels increases instantaneous transfer of glucose from capillaries to astrocytes and neurons, providing energy to support normal levels of firing even in the case of glycogen depletion.

**Figure 4:**
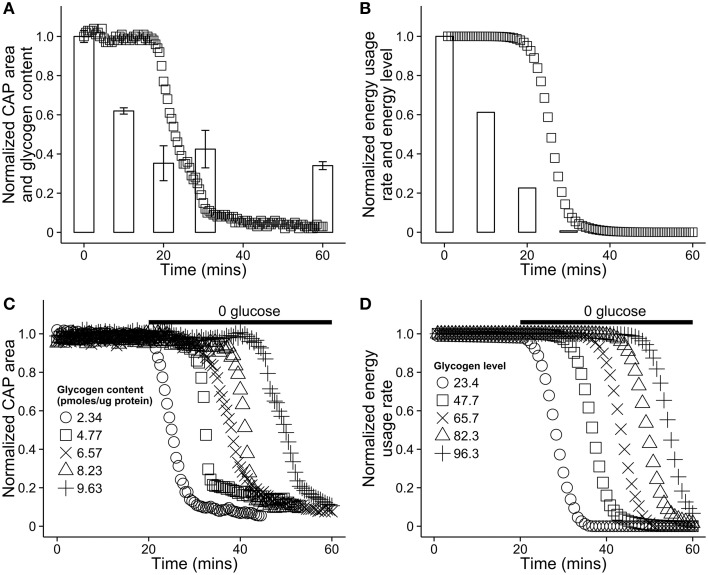
**Comparison of the energy dynamics of our model with the dynamics of astrocytic glycogen and compound action potential (CAP) area *in vitro* in a mouse optic nerve as reported in Brown et al. (2003). (A)** Recorded CAP area in a mouse optic nerve (squares) and glycogen content (bars). The nerve was stimulated in a solution with no glucose. Note that glycogen is not further depleted after reaching ≈25% of its original level. Figure recreated using data from Brown et al. (2003). **(B)** A simulation of the same situation as **(A)** using our model. To simulate aglycemic conditions, the energy flow from the capillary, *E*_*B*_ was set to 0 for the duration of the simulation. **(C)** Recorded CAP area in the mouse optic nerve *in vitro* as a function of time and the initial glycogen content in nearby astrocytes. At 20 min, the solution was replaced with a solution containing no glucose. Figure recreated using data from Brown et al. (2003). **(D)** A simulation of the same situation as **(C)** using our model. The absence of glucose in solution was modeled by again setting *E*_*B*_ = 0 at 20 min. In our model, astrocytic energy level ranges from 0 to 100, arbitrary units.

Glucose in the blood is treated as an infinitely large resource pool from which resources are extracted at a set rate. In principle, blood glucose could be modeled as finite pool larger than that of astrocytes. With the exception of Brown et al. (2003), few results exist detailing glycogen dynamics on the time scale of our model, and for this reason model parameters were selected to match glycogen dynamics results found in that paper (see Section 3 for parameter values). A comparison of our model dynamics in hypoglycemic conditions with mouse optic nerve data from Brown et al. (2003) can be seen in Figure 4. The figures highlight the effect of varying astrocytic glycogen content on the duration of sustained neural activity.

#### 2.3.1. Costs

Cognitive processes are called costly if they are processes to which individuals are generally averse. That certain cognitive processes are aversive is not in doubt—researchers have observed for decades that certain cognitive functions are aversive (see e.g., Tversky and Kahneman, 1974), necessitating in turn that humans exhibit a tendency to rely on habits (Redish, 2013) or heuristics (Gigerenzer and Goldstein, 1996) to perform tasks. Many suggestions have been made as to the nature of the cost driving cognitive aversion: for example that cognitive effort requires foregone leisure (Kool and Botvinick, 2014), that cognitive costs are a form of opportunity costs (Kurzban et al., 2013), or that costs are related to the need for extensive cognitive processing (Kool et al., 2010; McGuire and Botvinick, 2010).

Despite widespread agreement that cognitive effort is aversive, very little work has been done to quantify this aversion. We propose that a useful way to quantify aversion is in terms of foregone reward. The ability of incentives to induce task engagement is a standard operating assumption in experimental psychology and neuroscience. Extrinsic monetary or caloric reward is only one type of reward and is often compounded by and competes with other extrinsic (social, sexual) and intrinsic (curiosity, empowerment, mastery) rewards for impact on decision outcomes. However, it is easily quantified and has been shown to impact cognitive effort (see Camerer and Hogarth, 1999 for a review). We therefore restrict our treatment of reward to extrinsic reward for task performance (e.g., monetary), though other reward types could in principle be accounted for.

Reward and cognitive effort appear to balance in some way, resulting in a decision regarding whether or not to deploy effortful cognitive strategies. To our knowledge, only one group has attempted to quantify aversion to cognitive effort in terms of foregone reward. Braver and colleagues rewarded subjects for completing an *N*-back task (Westbrook et al., 2013). After an initial familiarization phase, subjects could make choices of whether to complete a more difficult task (higher *N*) for more money, or a less difficult task (lower *N*) for less money. Subjects were explicitly told that reward was contingent on maintaining effort rather than performance. In this way, the researchers effectively determined the amount of money subjects were willing to forego in order to avoid a cognitively demanding increase in *N*. They found that the subjective value of each level of *N*, or the amount by which increasing *N* decrease the preference for an offered reward, decreased linearly with the magnitude of *N*. These results are fully consistent with the additive objective function, although we suspect a more complex objective function will be needed as experimental results become rich enough to invalidate a first-order Taylor approximation. However, the deeper implication is that for difficult tasks, by suitably increasing the offered reward the subject will eventually overcome task aversion and attempt the task. For a recent review of neuroeconomic approaches to understanding cognitive effort, see (Westbrook and Braver, 2015).

#### 2.3.2. Controls

By treating cognitive effort as a resource control problem we are assuming the brain has mechanisms that allow control of cognitive effort and that varying effort affects subsequent reward. We believe there are limitations on both the ability to control resource allocation as well as limits on the impact that effort has on performance. Evidence suggests that cognitive effort can increase performance in certain tasks but not others. Camerer and Hogarth (1999) reviewed research investigating the relationship between financial incentives and performance in experiments. Their findings were nuanced. Higher incentives do not appear to improve performance in a wide range of task types, including tasks requiring insight (an “ah-hah! moment”). Incentives can consistently harm performance in a judgment and decision making tasks in which expert judgment can routinely be out-performed by a simple rule based on quantifiable observations. In this type of task, incentives appear to induce subjects to expend greater deliberative effort, weighting their own (inaccurate) judgements more highly than the predictions of formulas. To be clear, in these tasks incentives appeared to increase effort, but *not* objective performance. One simple way to reconcile these results is to assume the existence of both model-based high-cost deliberative neural computations and low-cost model-free experience-based paths (Daw et al., 2005), with the switch to deliberative decision-making resulting in a reduction in performance in cases where model-free solutions are superior.

In addition, incentives are most likely to improve performance in a subclass of judgment and decision making tasks, specifically tasks that are “effort-responsive.” These tasks critically depend on the cognitive functions which are the strongest candidates as controllable resources. In tasks where these functions operate, extrinsic motivation can “improve recall of remembered items, reduce the effect of anchoring bias on judgment, improve some kinds of judgments or predictions, improve the ability to solve easy problems, and also sharpen incentives to make zero-profit trades in auctions or do piece-rate clerical work” (Camerer and Hogarth, 1999). These tasks share a reliance on the use of executive function and working memory resources. For our purposes, the important finding is that in a specific but useful set of task types, monetary incentives can explicitly increase the utilization of the exact cognitive faculties that are considered cognitively costly.

There is currently insufficient data to enable us to identify a specific molecular mechanism for the proposed control. However, given the aforementioned connections between metabolic resource levels and offered reward on the one hand, and subject effort and performance on cognitively demanding tasks on the other, it is reasonable to posit the existence of some signal by which neuronal gain is modulated according to both the availability of metabolic resources and reward signals. One proposal for such a signal is some subset of neuromodulators associated with vigor acting on local excitatory neural gain via glutamate and glycine, though future research is needed to confirm this hypothesis. Botvinick and Braver (2015) provide a review of the possible neural mechanisms of cognitive control, and suggest dopamine as a candidate control signal.

#### 2.3.3. Model

The control variable in our model is a brain region-specific increase in neural excitability which we assume serves to recruit more neurons and consume more energetic resources. This control can also be understood as a requested size of a neural population dedicated to a particular task, with the request coming in the form of some excitatory neuromodulator. This control mechanism is compatible with modulation of attention (Nieuwenhuis and Yeung, 2005), and probably working memory, executive-function, including vigilance, and model-based lookahead size. However, we also acknowledge that there is an excellent case for an alternative mechanism for cognitive control by switching between decision making systems or policies that differ in costs and performance but can solve the same task. Many recent models of cognitive effort take this approach and simplify the graduation in cognitive effort into two categories of decision-making processes such as “System 1” and “System 2” (Stanovich and West, 2000), procedural and deliberative (Redish, 2013), or model-free and model-based (Daw et al., 2005) (for a review of dual-process models of decision making, see Evans, 2008). We believe a complete account of the control of cognitive effort will incorporate both of these control mechanisms (graded neural recruitment and strategy switching), a point we return to in the general discussion.

An important implication of interpreting the cost as something to be minimized *over time* is that agents optimizing Equation (1) are not myopic: an agent may choose an action that decreases an instantaneous reward in order to maximize reward in the long run. In the context of cognitive costs, there are two basic possibilities:

1. An agent avoids demanding cognitive processes because they are intrinsically costly. In this case the use of such processes would explicitly represented in the objective function. This is the approach implied by McGuire and Botvinick (2010) and elsewhere.
2. An agent generally avoids demanding cognitive processes *because doing so optimizes reward in the long run*.

The second option is analogous to the situation a runner finds herself in when competing in a marathon: sprinting at the beginning of the run is locally optimal but globally disastrous, because this strategy quickly exhausts the limited resources available. Analogously, it is possible that aversion to cognitively demanding processing is not a cost *per se*, but a strategic use of a limited glycogen reservoir. If this is the case, an aversion to certain cognitive processes may appear to indicate a cost but instead be a long-run performance-preserving strategy. In this case, cognitive costs might not belong in the objective function at all. Our approach encompasses both views, with zero λ parameter in Equation (5) for the long-run view, and positive λ for the intrinsic cost view. We explore the predictions made by each of these assumptions in the Section 3.

#### 2.3.4. Fatigue

In order to effectively utilize control actions, a controller must maintain an estimate of the current state. We propose that a key signal carrying the current energy level maps onto *subjective fatigue*. One might be tempted to suggest that mental fatigue and associated performance decrease is a consequence of resource depletion. Experimental findings contradict this, indicating instead that sufficiently high motivational incentives induce cognitive effort in spite of subjective fatigue (see e.g., Boksem et al., 2006).

We suggest that fatigue is not a signal of total resource depletion but of impending depletion if the current rate of use is maintained. In other words, subjects feel fatigued when glycogen stores anticipated to deplete at current usage, and fatigue is a relatively crude signal of partial depletion.

The link between subjective fatigue and partial glycogen depletion has experimental support (Matsui et al., 2011). Thus, fatigue serves as an important signal enabling subjects to estimate their energy state and plan their cognitive strategy accordingly. If immediate incentives warrant further use of a costly strategy, an individual may indeed temporarily continue its use. The implication by critics is that this should be impossible if a resources is in fact depleted and therefore unavailable for further utilization. If fatigue indeed reflects only *partial* glycogen depletion, this criticism becomes irrelevant. We would nevertheless predict that at a long enough time scale, individuals would become fatigued beyond the point of incentives to improve performance because the required resources simply would not be available. While this proposal suggests a global signal for resource depletion, we believe that cognitive fatigue has gradations across brain areas which may produce activity/task dependent fatigue.

## 3. Simulations

**Figure 5:**
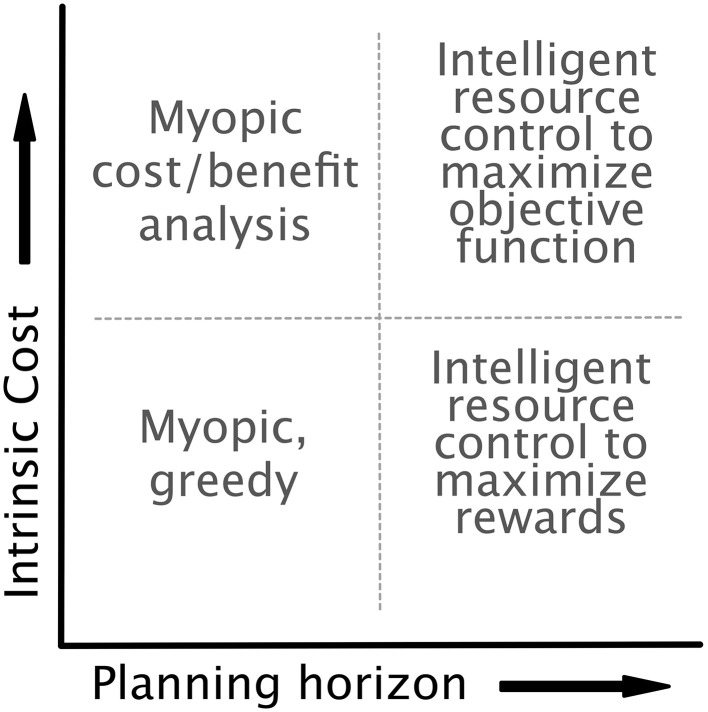
**The model is agnostic as to both the intrinsic cost of control (i.e., whether control is included in the objective function of the optimal control formulation) and the planning horizon of the agent**. This flexibility highlights the generality of the optimal control approach. Each choice of planning horizon and intrinsic cost results in an agent with a distinct optimization strategy.

We implemented our model with the dynamics, controls, and costs specified above. The optimal control framework allows (and forces) us to be explicit about an agent's lookahead and the components of the objective function. As mentioned in Section 2.3.3, it is unclear whether aversion to expensive cognitive processes is the result of their being intrinsically costly or simply an effect of long-term strategy. This ambiguity allows us to consider a space of models as indicated in Figure 5, with axes representing planning horizon and the intrinsic cost of cognitive effort. On the extremes of the planning axis, we can treat an agent as acting either myopically, with a decision based on only current costs and rewards, or optimally, with a lookahead equal to the time horizon of the task. On the intrinsic cost axis, we can omit costs arising from the cognitive control completely (low costs), or we can include control costs directly in the reward function (high costs). We assume for simplicity that the agent has error-free access to the current state and system dynamics, though in future work we intend to relax this assumption, as these errors may be critical for explaining behaviors that are essentially “illusions of cognitive costs.”

### 3.1. Implementation

Optimal control solutions were calculated using dynamic programming for the model in two experiment described below. In both cases the model was implemented with the values for constants listed in Table 2.

**Table 2:** **Constants used in the computational implementation of the model**.

**Name**	**Value**
Initial *H*_*N*_, *H*_*A*_	100
*a*_*connector*_	0.5
*a*_*A*_	10
*a*_*N*_	1
α, β	0.00042
δ	0.05
γ	0.02
γ_*e*_	6
γ_*f*_	5
*E*_*B*_	100

For an explanation of parameter values, see Section 2.2.1.

The dynamics used are listed in Equation (3). The objective function for each step *k* is given in Equation (5). Each dimension of the state space had a range [0, 100]. The state space was discretized to integer values. Transitions were probabilistic based on the fractional part of each dimension of the new state. For example, a value of 2.1 would be discretized as 2 90% of the time, and 3 10% of the time. At each time step, control levels were chosen from were chosen from *U* = [0.5, 1, 2, 3, 4, 5].

A control action was taken at each time step *k*. The temporal resolution for the dynamics model was higher than that of the control actions, so once a control action was selected, the system was run forward 100 iterations using Euler integration while maintaining the selected control.

The optimal control strategy was calculated using dynamic programming for a discrete state space as described in Bertsekas (2005). To represent the conditions described in Figure 5, simulations were performed for lookahead values of 1 and 100. In the first case, the control cost is simply treated as the single state's *g* function and the control is chosen according to

(6) $$\begin{array}{r} u_{selected}=argmin_{u\in U}g\left(x_{k},u\right) \end{array}$$

With a lookahead equal to the *N*, the control at each step is chosen recursively according to

(7) $$\begin{array}{r} u_{selected}=argmin_{u\in U}\left\{g_{k}\left(x_{k},u\right)+J_{k+1}\right\} \end{array}$$

where *J*_*k* + 1_ is the minimum cost-to-go from step *k* + 1 onward.

### 3.2. Simulation 1

In the first simulation, a finite time horizon of *N* = 100 was used and reward (equal to performance) was assumed to be available for the duration. Optimal control trajectories were calculated for lookahead values of 1 and 100 and for λ = 0, 0.1, 0.4. The value λ = 0 reflects the case in Figure 5 in which cognitive costs are not included in the objective function. As discretization of the state space led to stochastic state transitions, simulations were run four times and results were combined using LOESS smoothing.

**Figure 6:**
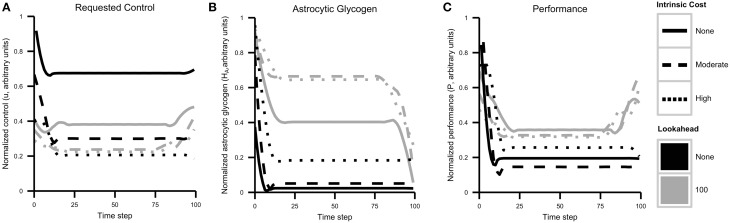
**Cognitive effort avoidance can be explained as resource allocation with a lookahead**. Plots show results from Simulation 1, separated by agent lookahead and intrinsic cost (see also Figure 5). **(A)** Computed optimal control *u* over time. The highest level of control is requested by an agent with no lookahead and no intrinsic cost of control. Note that actual performance depends both on requested control and energy availability. **(B)** Glycogen energy levels *H*_*A*_ for each case. Note that agents with no lookahead deplete glycogen much more quickly. **(C)** Agent performance over time. As expected, agents with a longer lookahead and lower intrinsic costs exhibit better overall performance while generally exercising less control (less neuromodulation).

Control, resource, and performance trajectories for each combination of lookahead and λ are shown in Figure 6. The simulation results reveal several important patterns. First, a higher control level is sustained when cognitive costs are not included in the objective function λ = 0. In addition, control strategies that include a lookahead of 100 maintain glycogen levels that are much higher than those maintained by no lookahead. This result reflects strategic resource allocation over time, a feature that is not present with the agent cannot foresee system dynamics in the longer term.

The most important result relating to the discussion above is that when a lookahead is included, the optimal solution is to sacrifice performance initially in order to maximize overall performance. Comparing the initial requested level of control (that is, allocation of neural resources) and resulting performance with the long-term performance curve, one is again reminded of a marathon runner. Without the ability to predict the depletion of resources and its effect of resource depletion on performance, the agent initially requests a high amount of resources. This quickly decreases the level of astrocytic glycogen, which leads to a lower sustainable performance level and overall lower performance.

To rephrase this result in terms used above, what appears to be an initial aversion to cognitive costs is actually a strategic decision to avoid resource depletion and maximize long-term reward.

### 3.3. Simulation 2

In the second simulation, reward was made unavailable for certain periods, simulating conditions where no reward is available to the agent (resting between experimental tasks, for example). The dynamics, parameters, and objective function were identical to those used in Simulation 1, with the exception that the performance function returned 0 for two periods of time, regardless of energy level or control action. The simulation was run five times for 500 steps, and again the results were combined using LOESS smoothing.

**Figure 7:**
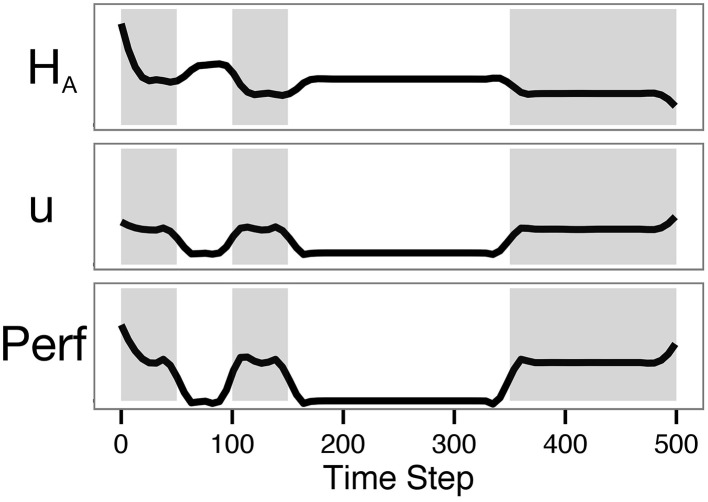
**The model predicts lower performance on the second task of a dual-task paradigm**. The plot shows results from Simulation 2, with zero intrinsic cost and a lookahead of 500. Shading indicates periods in which the agent could be rewarded (on-task periods). Note that overall performance in the second shaded period is lower than performance in the first.

Simulation traces are shown in Figure 7. Though control was not incorporated in the objective function, the control action was nonetheless moderate throughout the simulation. Control was minimal during the no-reward periods, as expected. That the control does not appear to be a step function at the transition points is due to the smoothing method used. As seen in Baumeister's dual-task ego-depletion experiments, performance was highest in the first rewarding period and diminished in the second. Though astrocytic energy levels rebounded somewhat during rest periods, the requirement of a minimal level of control (*u* = 0.5 rather than *u* = 0) meant that available energy was never fully replenished. This is in accordance with evidence suggesting that astrocytic glycogen is slowly depleted during waking hours and substantially replenished during a few hours of sleep (Karadzic and Mrsulja, 1969; Swanson, 1992).

### 3.4. Model extensions

As presented here, our model treats energetic resources as an energy flow from capillaries to astrocytes and neurons. The simulations above provide qualitative relationships between control, performance, and energy dynamics. It would not be difficult to calibrate the model in order to match known rates of glucose transfer from capillaries to astrocytes and neurons. The energy flow could be changed to use diffusion equations instead of liquid flow equations. Conversion of glucose to glycogen then lactate is somewhat inefficient, and that inefficiency could be included in the model. The agent's state is currently assumed to be perfectly known by the agent, but a more realistic approach would be to have the agent estimate its state from a crude, noisy input. This would reflect our suggestion that fatigue is a signal of partial glycogen depletion. These complicating factors were omitted from the current model to preserve as much simplicity as possible while still providing a useful conceptual framework. Even so, the model already provides qualitatively useful predictions (see e.g., the astrocytic glycogen levels in Figure 6B, the performance trajectories in Figure 6C, and the presence of a performance deficit in subsequent tasks in Figure 7).

### 3.5. Testable predictions

Given the model outlined above, there is a way to test whether the cognitive cost parameter λ is greater than 0, that is, whether the objective function in our model should include a penalty for cognitive control. The planning-based model introduces a tradeoff between current and future resource utilization, with no particular penalty for current utilization over future. In contrast, a cognitive cost of λ > 0 implies a trade off between costs and rewards in the present with no consideration of the future. These models make different predictions in a condition where a time horizon is short or non-existent (in other words, in which no planning is needed). One way to induce such a scenario is to provide an immediate and abundant source of energetic resources, such that high utilization of astrocytic glycogen is not necessary, even in conditions of high neural activity. To do this, one could clamp an individual's blood sugar to a high level, such that all additional energy utilization arising from demanding cognitive processing is provided from the blood (the capillary-neuron connection in Figure 1B) rather than the astrocytic glycogen shunt. In this condition there are no future energetic consequences to high neural activity. Our model predicts that in such a condition, subjects should not show cognitive cost effects if λ = 0, in other words, all cognitive effort aversion can be attributed planning. If cognitive cost effects persist we would conclude that λ > 0, meaning that cognitive effort should be treated as a cost *per se*.

The addition of a glycogen energy store allows us to make a prediction that would potentially falsify either our account or the glucose-only “limited resource” account. Consider a set of tasks arranged in the commonly used dual-task paradigm in which the first task is cognitively demanding enough to negatively affect performance on the second task. As mentioned, several groups have reported that the consumption of a glucose drink negates the negative effect of performing the first task (Manning et al., 1990; Benton and Owens, 1993; Craft et al., 1994; Benton and Parker, 1998; Martin and Benton, 1999). Suppose the first task is performed and then a glucose drink is administered. Now consider a manipulation in which blood sugar is returned to the pre-task level, e.g., via insulin injection, and then subjects are asked to perform the same task again. If astrocytic glycogen indeed acts as an energy shunt that is depleted during the task, as we suggest, glycogen levels should still be largely depleted following insulin injection. Because of this, performance on the task should be *worse* than initial performance, despite equivalent blood sugar levels. If, on the other hand, glycogen does *not* act as an energy shunt and all energy is taken directly from blood glucose, performance on the task should be equivalent both times. Note that this prediction only holds in cases where there the first task produces a reliable performance deficit in subsequent tasks.

## 4. Implications

The idea that the brain has a sophisticated controller for resource allocation has a number of important implications that we believe provide a novel explanatory synthesis of a range of phenomena. The timescale of the dynamics of glycogen depletion puts limits on sustained neural activity and introduces potentially costly consequences to operating at peak performance in one task on the performance of subsequent tasks. We term controlling resource allocation to optimize long-term performance *optimally lazy*, which amounts to incorporating future resource availability into decisions about how to recruit neural activity to solve a particular task in the near-term. Although we developed our model around the control of neural recruitment, we believe that there are probably at least two additional and important control schemes that operate at higher spatial and temporal scales. The putative control schemes include:

### 4.1. Control by modifying gain on neural activity

This control mechanism is the focus of our paper. It optimizes energy consumption on the timescale of an neural population that has shared glucose dynamics and could be the target of a local neuromodulator gain control signal. This control strategy provides a new role for excitatory and inhibitory neuromodulators, and we believe it includes known gain control systems like attention, working memory, and executive function. We expect to see this type of resource control at the intersection of neural processes that are costly and whose recruitment makes a graded impact on performance. While we modeled these gains at the level of a neural population, it is more likely that gain control is structured hierarchically, with multiple levels of resolution. For example, the concept of *vigor* can be implemented by a more global gain on goal-oriented behavior. Vigor is known to be modulated by resource availability, with rich resource availability increasing willingness to work in humans and animals, and in the speed and variety of behaviors expressed (Niv et al., 2005).

### 4.2. Control by switching strategies or policies with different performance/efficiency trade-offs

There are also advantages to incorporating knowledge of differential costs of neural activity to learn more metabolically efficient strategies or policies. Investments in efficiency have the largest pay-offs in frequently recurring tasks. If model-based look-ahead is indeed differentially costly, then efficiency may drive model-free policy learning. The idea that efficiency drives model-free learning is a significant change of viewpoint. Model-free methods are usually justified on the basis of improved accuracy as experience grows (Daw et al., 2005; Fulvio et al., 2014). However, congruent with efficiency driving learning, Huang et al. (2012) provide evidence that suggests later stages of motor learning are directed toward increasing metabolic efficiency, without gains in performance. More generally, varying the weight on metabolic costs during task learning can produce a family of solutions that can be rapidly selected between during a task to provide an alternative online control of metabolic costs. Given a family (at least two) strategies (policies) that have different neural costs and performance on the same task, a controller can select (or weight) policies to trade-off performance vs. cognitive costs. The current model allows for this possibility via the inclusion of multiple performance curves, as seen in Figure 3.

### 4.3. Control by modifying the distribution of glycogen across the brain

When efficient solutions to recurrent tasks are not available (or take too long to effectively learn), the brain may invest in changing the distribution and quantity of energetic stores across the brain, either by changing locations and numbers of astrocytes and/or the amount of glycogen stored in astrocytes. Reallocation of energetic stores constitutes a distinctive kind of control that serves to anticipate and meet the demands of recurrent neural activity at longer timescales. If present, it would constitute a distinctive type of *endurance learning*, that would provide a basis for generalizable gains in resource dependent cognitive processes like attention, working memory, look-ahead, and executive function. It is also strongly analogous to changes in muscle glycogen stores induced by exercise. Consistent with this idea, hypoglycemia (low energy availability) causes lower cognitive/physical performance, and we have a real need for rest after both heavy cognitive exertion similar to physical exercise. The cognitive benefits of physical exercise may result in part from better metabolic regulation, and cognitive training may produce increases in glycogen similar to the impact of physical training on muscle glycogen. Congruent with this possibility, the time scale of glycogen repletion in the brain is similar to that in muscle also overcompensation afterwards (Matsui et al., 2012), and glycogen depletion leads to super-compensation of glycogen levels in astrocytes (Choi et al., 2003). Changes in resources may underlie the pattern of cognitive gains documented in video game players, which show general improvements in visual attention and working memory (Bavelier et al., 2012), while simultaneously providing a fundamental reason for the ubiquitous finding of lack of transfer in most learning paradigms—learning paradigms are almost universally conducted in conditions where task recurrence would encourage the investment in efficient (but necessarily specialized) solutions.

Considering glycogen allocation as an optimal resource allocation problem, we predict that the differential density of astrocytes and glycogen stores across the brain will be a monotonic function of the frequency of sustained activity. Areas whose activity are infrequent or short relative to depletion dynamics will need less resources than areas whose activity is frequent and sustained. This relationship between the statistics of sustained neural activity and glycogen stores are easily testable. In general, this predicts that the more specific the cognitive function, the less frequent its sustained use and the more susceptible it will be to degradation with decreases in energy availability. As sustained functions are likely recoded in efficient ways to exploit more direct sensory to motor mapping, we predict this degradation will fall most heavily on higher cognitive functions. The best evidence for this idea are the patterns of loss of cognitive with declines in blood sugar, in which working memory, attention, and executive control are more sensitive to hypoglycemia than visual and auditory acuity or basic motor functions (Feldman and Barshi, 2007).

### 4.4. A mechanism for the integration of energy utilization

The model presented in this paper requires some mechanism by which the depletion of astrocytic glycogen is translated into effort avoidance behavior. Accordingly, one prediction of our model is that the trajectory of glycogen storage levels should be coded neurally at a larger scale than local negative feedback due to neurotransmitter concentrations. This energy usage information must then be transmitted to structures controlling cognitive effort expenditure in order for it to manifest as behavior. We do not make a specific claim about the identity of this circuit, but the evidence presented in the review above, combined with the explanatory power of the model presented in this paper, strongly suggest that it exists.

In conclusion, by viewing the allocation of metabolic resources as a control problem with the concrete resource limitation given by the dynamics of glycogen storage and use, we provide a family of novel explanations for a number of apparently unrelated phenomena while simultaneously providing a rational explanation for a wide range of troublesome biases and patterns in decision making. These new hypotheses are quantitative, testable, and we hope will provide grist for the development of new explanations and interventions.

## Author contributions

Both authors performed the literature review, developed the theoretical framework, and wrote the paper. TC performed the computational implementation of the model and the relevant simulations.

### Conflict of interest statement

The authors declare that the research was conducted in the absence of any commercial or financial relationships that could be construed as a potential conflict of interest.
